# Secondary imperfectivisation is reverbalisation

**DOI:** 10.1007/s11525-025-09440-7

**Published:** 2025-05-16

**Authors:** Boban Arsenijević, Stefan Milosavljević, Marko Simonović

**Affiliations:** University of Graz, Graz, Austria

**Keywords:** Serbo-Croatian, Theme vowel, Secondary imperfectivisation, Verbalisation

## Abstract

We argue that secondary imperfectivisation in Serbo-Croatian (and possibly in Slavic more generally) is a procedure whereby a new verb is derived from a perfective verb, i.e., the perfective verb gets reverbalised. This operation takes a perfective verb, that is, a verbal structure including an aspectual projection that restricts its interpretation aspectually (but also adds some lexical semantic content) and turns it into a bare *v*P. The new verb preserves the conceptually enriched semantics, but not the structurally derived aspectual restriction, which gets overwritten by the newly projected category. The inner aspect of the verb is thus reset to the unvalued, i.e., aspectually unrestricted default. The verb gets interpreted as imperfective by scalar implicature: had the speaker intended the perfective interpretation, they would have used the more specific and computationally cheaper expression involving the perfective verb. On the morphophonological side, assuming with a host of literature that the verbal category is realised by theme vowels (Spyropoulos et al., 2015 and Panagiotidis et al., 2017 for Greek, Svenonius, 2004a for Russian, Jabłońska, 2004, 2007 for Polish, Biskup, 2019 for Czech, Milosavljević & Arsenijević, 2022 for Serbo-Croatian), imperfectivisation amounts to stacking theme vowels. Support for the view comes from Simonović et al. (2023), who arrive at the same conclusion on morphophonological grounds.

## Introduction

The main claim of this paper is that the derivational process in the domain of the Slavic verb commonly referred to as secondary imperfectivisation should be reanalysed as reverbalisation: a process whereby a new verb is derived from an existing perfective verb. While the input verb involves a structurally derived aspectual restriction, the new verb fits a bare *v*P, where the category head renders the aspectual specification of its perfective base inaccessible for interpretation. This semantically resets the interpretation of inner aspect from the restricted, perfective value, to the unspecified, unrestricted value, while preserving all the lexical semantic content added by the respective structure within the base. The imperfective, i.e., atelic interpretation of the result emerges via scalar implicature (Horn, 1984), i.e., via antipresupposition (Percus, 2006): the choice of the more complex, less semantically specific alternative infers that the meaning of the simpler and more specific verb was not intended. Our data come from Serbo-Croatian.

The paper is organised as follows. In the remainder of the introduction we first present secondary imperfective affixes in Serbo-Croatian and summarise a previous proposal which, based mainly on morphophonological facts, analyses secondary imperfectivisers as consisting solely of theme vowel material (Section 1.1), and then outline the core idea of this paper (Section 1.2). In Section 2, we review the key concepts relevant to the paper: different approaches to telicity and grammatical aspect (Section 2.1), as well as previous approaches to secondary imperfectivisation (Section 2.2). Our proposal is presented in Section 3. Section 4 concludes the paper.

### Secondary imperfectivisers in Serbo-Croatian

Secondary imperfectivisation is typically defined as a process whereby a verb previously rendered perfective (in Slavic, typically by prefixation) becomes imperfectivised (in Slavic, typically by suffixation),^1^ as illustrated in Table 1.^2^

**Table 1:** The typical three-step derivation of secondary imperfectives in Serbo-Croatian

‘simplex’ ipfv	prefixed pfv	secondary ipfv
‘ask’	‘examine’	‘examine’
‘build’	‘partition’	‘partition’
‘write’	‘copy’	‘copy’
‘read’	‘project’	‘project’
‘empty’	‘vacate’	‘vacate’
‘plough’	‘rummage through’	‘rummage through’

Table 1 contains Serbo-Croatian examples of the typical three-verb chain, whereby the first member is an imperfective verb which has the minimal structure root+theme vowel+inflectional morphology, in this case the inf morpheme . The verbs in the first column in Table 1 illustrate the three most common theme vowel classes in Serbo-Croatian, accounting for more than 70% of all verbs (Arsenijević et al., 2024): a/a^3^ (illustrated by  and ), i/i (illustrated by  and ) and a/je (illustrated by  and ). The verbs in the second column in Table 1 are the respective prefixed counterparts, which typically have a shifted lexical meaning with respect to the base verb. The verbs in the third column are secondary imperfectives, which keep the meaning of the perfective verbs but shift the aspect class to imperfective.

The underlined affixes in Table 1 are so-called secondary imperfectivisers. The exemplified secondary imperfectivisers,  and , are also the two most frequent secondary imperfectivisers in Serbo-Croatian. There are pressing arguments against the simplest possible analysis implied by our representation in Table 1, i.e., against assuming // and // as direct exponents of the feature [(sec)ipfv]. First, both affixes sometimes co-occur with the palatalisation of the preceding consonant, as attested by the roots // and //, which surface as [] and [] in the examples above. This led authors of traditional analyses to postulate four affixes //, //, // and // (see, e.g., Babić, 2002, 526). Second, and more importantly, the sequence [] does not surface as such in all forms of secondary imperfectives. It shows up as [] in the present tense and in a set of other forms, as shown in Table 2.

**Table 2:** ∼ alternation in Serbo-Croatian secondary imperfectives

inf	prs.1pl	gloss
		‘examine’
	-mo	‘partition’

This allomorphy pattern is not just an argument for listing multiple allomorphs of the morpheme , but also for further decomposition of  and other secondary imperfectivisers. The two allomorphs []∼ can be decomposed into the exponents of the theme vowel /, illustrated by, e.g., the verb ∼ (from underlying //) ‘to write ∼ we write’, plus a residue. Segmenting ∼ as ∼ (and, by the same token, ∼ as ∼) is therefore a plausible next step. This analysis has been proposed by Quaglia et al. (2022), who argue more generally that Slavic secondary imperfectivisers can be viewed as ‘mini verbs’ (i.e., verbs without lexical semantics) contributing their own theme vowels, which surface to the left of inflectional morphology. This is based on the observation that “secondary imperfectivisers, at least in Slovenian and B/C/M/S, end in a theme vowel”.^4^

The same observation has been made by Marković (2018, 78) for several common secondary imperfectivisers and a dozen other verbal affixes in Serbo-Croatian. Where the analyses in Quaglia et al. (2022) and Marković (2018) differ are the residual sequences ∼ and ∼. Quaglia et al. (2022) assume that these are bound roots which can display phonologically unpredictable root allomorphy, comparable to that in ∼ (from underlying //) ‘to send ∼ we send’ or ∼ (from underlying //) ‘to slaughter ∼ we slaughter’. Marković (2018), on the other hand, assumes underlying representations of  ∼  and ∼ which only consist of vowels, i.e., ∼ and ∼ respectively. He further assumes that the segments  and  are inserted by a morphologised process which removes hiatus.

Simonović et al. (2023) follow Marković’s approach and take it even further, arguing that secondary imperfectivisers consist solely of theme vowel material (similar to Feinberg (1980)’s and Enguehard (2017)’s analyses of Russian verbal suffixes). Apart from the secondary imperfectiviser-final theme vowel (e.g., ), this then also includes the hiatus-preventing consonant (e.g., ), and the vowel preceding this latter consonant (e.g., ). Finally, the palatalising feature that sometimes occurs on the left edge of the traditional secondary imperfectivisers is also analysed as theme vowel material. For instance, the secondary imperfective  ‘vacate.ipfv’ (related to the prefixed perfective  ‘vacate.pfv’) is analysed as preserving the original theme vowel in the form of a palatalising feature. Following the proposal in Simonović et al. (2023), the underlying representation of  would be / /, where the superscript marks floating material^5^ and the exact output is determined by phonological constraints. In a detailed overview of the imperfectivisation patterns, Simonović et al. (2023) show that other, less productive Serbo-Croatian secondary imperfectivisers can be analysed in terms of theme vowel stacking as well.

### Proposal in a nutshell

Against the presented background, the central question is why (sequences of) theme vowels are employed to achieve (secondary) imperfectivisation. Assuming that theme vowels are signs of verbalisation, imperfectivisation would then amount to reverbalisation (or even re-reverbalisation). The main consequence of imperfectivisation reanalysed as (re-)verbalisation is that imperfective verbs are semantically and syntactically unspecified and thus compatible with all kinds of eventualities, both completed and incompleted. Perfective verbs, on the other hand, contain a syntactic feature that restricts this set of eventualities only to those satisfying telic descriptions (roughly, completed events).

Our core insight can be implemented in any approach that assumes the presence of syntactic features in morphology. The specific implementation presented here, however, assumes categories that are separate from roots, as is generally assumed in, for example, Distributed Morphology (Halle & Marantz, 1993).

Analyses that treat verbal theme vowels as more than just empty ornamental elements most often analyse them as verbalisers – whether as realisations of the verbal category, as in Spyropoulos et al. (2015) and Panagiotidis et al. (2017) for Greek, Svenonius (2004a) for Russian, Jabłońska (2004, 2007) for Polish, Biskup (2019) for Czech, Milosavljević and Arsenijević (2022) for Serbo-Croatian, or as light verbal roots, as in Fábregas (2017). The observation that secondary imperfectivisers actually present (sequences of) theme vowels then leads to the conclusion that secondary imperfectivisers function as reverbalisers.

As for the inner aspect specification, a bare *v*P is aspectually unspecified, hence refers unrestrictedly both to eventualities satisfying telic and strict non-telic predicates. The higher structure may or may not introduce a telic specification. If it does not, a traditional imperfective verb emerges. As a rule, a secondarily imperfectivised verb has a minimal pair, with equivalent lexical semantics, distinguished only by an aspectual restriction (i.e., by being a perfective verb). This triggers a scalar implicature that the meaning of the simpler, aspectually restricted verb was not intended (else that simpler and semantically more specific verb would have been used), narrowing down the interpretation from unspecified to what is traditionally described as imperfective.

We follow Borer (2005), Łazorczyk (2010), Arsenijević (2023), Milosavljević (2023a,b) in analysing the opposition in Slavic traditionally captured in terms of perfective and imperfective verbs as one concerning telicity rather than imperfectivity. Combining this assumption with the approach outlined above, traditional imperfectives are then unspecified for telicity, including in their extension both eventualities that satisfy telic verbal predicates and those that do not, while traditional perfectives are specified as telic, and hence restricted to only certain eventualities. The higher structure (which may lack phonological realisation, or be realised by forms like the aorist, the imperfectum or the pluperfect) then specifies grammatical aspect (the relation between the event time and the reference time), including properties of the reference time such as collective or distributive plurality (leading, respectively, to iterative and habitual meanings). For both semantic and pragmatic reasons, which we do not discuss here (but see Arsenijević, 2013b), perfective viewpoint aspect tends to combine with telic verbs, and imperfective with atelic ones. With the cited approaches, we postulate a syntactic marking of telicity located in a functional projection right above the verbal category head, labeled in the literature in various ways, as QP, #P or (Inner)AspP. Structurally, thus, traditional imperfectives lack a low aspectual projection, for which we choose the label QP, while traditional perfectives have this projection filled with some relevant material. Following this view, we take the structure without QP to derive interpretations unrestricted for lexical aspect, of the kind attested in the traditional biaspectual verbs, i.e., we preclude the existence of imperfective verbs proper. As explained above, the reason why most traditional imperfectives under normal circumstances appear not to be satisfied by eventualities that satisfy telic predicates lies in a scalar implicature. Whenever a verb unspecified for telicity has a minimal pair which differs only in being telic, the hearer infers from the fact that the telic verb was not used that the speaker did not intend an eventuality that satisfies the telic counterpart (else, following the Gricean maxim of quantity, they would have used the more specific telic variant).

We assume with Borer (2005), Arsenijević (2006), Łazorczyk (2010), a.o., that telicity maps directly to the presence or absence of (the relevant material in) the QP in the verbal extended projection. The introduction of such a QP restricts the predicate to telic interpretations. Crucially, this step cannot be reverted: a specification can only be added, not removed. However, QP often contains material that contributes lexical semantics to the predicate, thus deriving new event descriptions. In consequence, those richer semantic descriptions remain unavailable for reference to eventualities which do not satisfy the telicity restriction.

To overcome this obstacle, grammar resorts to reverbalisation: it merges the derived telic structure with the category head *v* and thus derives a new verb which does not include a QP in its extended projection. The extended projection relevant for the interpretation is now the newly projected one: the aspectual interpretation is computed from there. This overrides the telicity restriction of the embedded verbal predicate, while preserving the rich description derived by it.

This analysis provides a way of accounting for the otherwise puzzling observation with which we began this section: secondary imperfectivisers in Serbo-Croatian, and possibly in other Slavic languages as well, can be analysed as (sequences of) theme vowels. Theme vowels realise the verbal category, and it is the verbal category that effects imperfectivisation.

Importantly, the reverbalised structure is atelic in the same way as simple atelic verbs: it is a bare *v*P, underspecified for lexical aspect, meaning its extension includes both atomic and non-atomic (mass) eventualities that satisfy the rest of the description. The stronger tendency of secondary imperfectives to have certain interpretations, compared to simple atelic verbs, arises from pragmatics. Secondary imperfectives are morphologically more complex than their telic bases, so their use more strongly triggers a scalar implicature, as they are employed in contexts where a simpler alternative was available. Simple imperfectives sometimes lack minimal perfective pairs (in which case they often behave as biaspectuals), and when such perfective pairs do exist, they tend to be morphologically more complex.

## Key concepts and previous approaches

### Telicity and grammatical aspect

Virtually all previous approaches to secondary imperfectivisation relate the core nature of secondary imperfectivisers to aspectual phenomena in one way or another. Many authors propose that secondary imperfectivisers are operators or exponents of the grammatical aspect, i.e., they mark viewpoint imperfectivity (e.g., Smith, 1997, Svenonius, 2004a,b; Ramchand, 2004, 2008; Borer, 2005; Progovac, 2005; Borik, 2006; Altshuler, 2013, 2014; Minor et al., 2022, a.o.), while for others secondary imperfectivisers are markers of atelicity or other notions of inner aspect (e.g., Klein, 1995; Łazorczyk, 2010; Ramchand & Minor, 2019; Kwapiszewski, 2020; Arsenijević, 2023; Milosavljević, 2023b). There are also approaches that indirectly link secondary imperfectivisers to grammatical aspect, by proposing that secondary imperfectivisers are hosted in some projection below grammatical aspect, yet they have either an imperfective feature, or a relevant semantic property that ensures that they are selected by the imperfective operator/head (e.g., Klein, 1995; Tatevosov, 2015, 2018; Mueller-Reichau, 2020; Biskup, 2023b,a). The overall landscape of analyses gets additionally complicated by the fact that definitions of grammatical aspect and its realisational possibilities (i.e., the imperfective, perfective, neutral aspect, etc.) originate from different approaches, so that this notion sometimes partly or completely overlaps with telicity, which itself also lacks a unified definition. To minimise vagueness and make the overview of different analyses of secondary imperfectivisers more comprehensible, we first briefly introduce the notions of telicity and grammatical aspect (see also Arsenijević et al., 2013; Arche, 2014a,b; Rothstein, 2016; Truswell, 2019; Milosavljević, 2023b for recent overviews).

**The value of telicity** (lexical aspect, inner aspect, situation aspect) has been approached in different ways in the literature – in terms of the event-argument homomorphism (Dowty, 1991; Krifka, 1992, 1998), the result state component (Pustejovsky, 1995), atomicity (e.g., Rothstein, 2004, 2008a,b), event maximalisation (Filip, 2004, 2008; Filip & Rothstein, 2006), singularity (Arsenijević, 2023), non-homogeneity/quantity (e.g., Borer, 2005), scale features (e.g., Hay et al., 1999). In this paper, we assume that the computation of telicity is based on the quantity properties along the lines of Borer (2005): a predicate is telic (= quantity) if it is non-homogeneous, i.e., if it is quantised or non-cumulative (see Section 3 for our more specific approach to telicity). In syntactic approaches, telicity is specified in the quantity projection immediately above the *v*P, i.e., AspQ (Borer 2005), QP (Arsenijević, 2006, 2007, 2013a, 2023; Milosavljević, 2023a,b), (Inner)AspP (Pereltsvaig, 1999, 2000; MacDonald, 2008; Travis, 2010; Łazorczyk, 2010). In those approaches that keep these projections apart (see also Harley, 2013), QP is usually sandwiched between the *v*P (= the verbalising projection) and the VoiceP (the projection hosting the external argument) (e.g., Pereltsvaig, 2005; MacDonald, 2008). Another prominent option in syntactic approaches to telicity is that it is computed at the level of *v*P, and the unique dedicated aspectual projection is the one hosting grammatical aspect (e.g., Ramchand, 2004, 2008; Svenonius, 2004b,a).^6^

The temporal modification test is the most standard diagnostic for (a)telicity, since it is employed regardless of the exact way telicity is approached. According to this test, durative adverbials, often referred to as *for*-adverbials, modify atelic predicates, whereas time-span adverbials, widely known as *in*-adverbials, modify telic predicates – but not vice versa, as in (1) from English.

1.
  - John ran *for an hour* / *in an hour. [atelic]
  - John wrote a letter *in an hour* / *for an hour. [telic]

The notion of **grammatical aspect** (also referred to as outer or viewpoint aspect)^7^ has also been approached in different ways. According to a wide-spread definition, imperfective viewpoint arises when the reference time is included in the event time, whereas perfective viewpoint stands for the event time interval being contained within the reference time interval (Reichenbach, 1947; Klein, 1994, 1995; Bhatt & Pancheva, 2005; Łazorczyk, 2010, among many others). In applying this basic definition to Russian aspect, Borik (2006) additionally includes the relation between the speech time and the reference time as a part of definition: besides the event time being contained in the reference time, the perfective aspect does not allow an overlap between the speech time and the reference time (i.e., S ∩ R = ∅ & E ⊆ R). The imperfective aspect is defined as non-perfective (¬ (S ∩ R = ∅ & E ⊆ R), i.e., S ∩ R ≠ ∅ ∨ E ⊈ R) (Borik, 2006, 187). In syntactic approaches that assume this view of grammatical aspect, the projection hosting it is usually taken to be generated between the *v*P (or the VoiceP, when these projections are kept apart) and the TP (cf. Klein, 1994; Pereltsvaig, 2005).

Ramchand (2004, 2008) sees grammatical aspect as an analog of definiteness/specificity in the verbal domain, with the perfective aspect marking definite/specific reference time, and imperfective aspect being a reflex of an indefinite reference time. The definiteness here is related to a specific point with respect to the run time of the event, and not to a specific moment on a timeline with respect to the utterance time (Ramchand, 2008, 1705). Demirdache and Uribe-Etxebarria (2014) analyse grammatical aspect in terms of anaphora resolution within the verbal domain. They claim that when temporal anaphora between the event time and reference time is resolved via binding, the resulting aspectual viewpoint is imperfective, while when it is resolved via coreference, the resulting viewpoint is perfective.

Before we proceed to alternative definitions of grammatical aspect, note that in the majority of approaches employing the relation between the event time and reference time, finiteness figures as a *conditio sine qua non* for licensing the aspectual projection (see especially Klein, 1994, 1995; Tatevosov, 2018). This is an important argument for separating Slavic verbs and their morphology from grammatical aspect (see Section 2.2.2). One of the tests that differentiates between imperfective and perfective viewpoints is the time interval test. Łazorczyk (2010) points to this test as one of the very few reliable ones that are not obscured by the interaction of (a)telicity and (im)perfectivity. Under the temporal interval test, a time-frame adverbial specifies the reference time. Predicates that, combined with a time-frame adverbial, only have the inclusive interpretation (i.e., the event time is properly included in the reference time) are perfective, whereas others are imperfective, as illustrated in (2) from Łazorczyk (2010, 55).

(2)
  - Between 10 and 11, Maya read a book and slept. (telic perfective [inclusive])
  - Between 10 and 11, Maya read and slept. (atelic perfective [inclusive])
  - #Between 10 and 11, Maya was reading a book and sleeping. (telic imperfective [intended: inclusive])
  - #Between 10 and 11, Maya was reading and sleeping. (atelic imperfective [intended: inclusive])

Another prominent line of analysis of grammatical aspect (with special emphasis on Slavic languages) is based on partitivity (e.g., Comrie, 1976; Smith, 1997; Filip, 1993, 2017; Altshuler, 2013, 2014). A classical definition, often assumed in the Slavic literature, is that by Comrie (1976, 16), for whom “perfectivity indicates the view of a situation as a single whole, without distinction of the various separate phases that make up that situation; while the imperfective pays essential attention to the internal structure of the situation”. According to Smith (1997, 66), the perfective viewpoint presents a situation as a whole, including initial and final points of the situation. The imperfective viewpoint, on the other hand, focuses on a part of a situation, with no information about its endpoints (*idem*: 73). For Filip (1993, 2017) and Altshuler (2013, 2014), imperfective and perfective operators are also partitive operators. However, unlike for Smith, in their approaches, the imperfective operator is compatible with any part (or stage) of a situation (including initial and final ones), i.e., it is compatible with both unbounded and bounded predicates. The perfective operator, on the other hand, requires proper event parts (in Filip’s terminology) or stages (in Altshuler’s terminology) in the denotation of the *v*P it combines with, i.e., it requires maximal parts/stages (roughly, it combines only with bounded predicates). The phasal-verbs test, which is often applied in the literature on Slavic languages, reflects the ‘partitivity’ view of aspect. According to this test, imperfective verbs, but not perfective verbs, can be used as complements of phasal verbs such as begin/continue/finish, as in (3) from Serbo-Croatian.

(3)
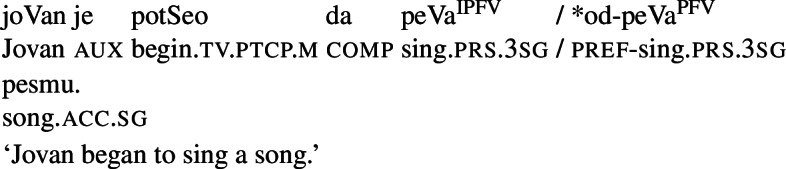

While in many approaches cited above both perfective and imperfective viewpoints/aspects are positively defined, i.e., they are separate operators and/or have different syntactic positions, there are also works in which imperfectivity is defined negatively in relation to perfectivity – i.e., as the unmarked aspect, the neutral aspect, the default aspect, the underspecified aspect, etc.,^8^ emerging in the absence of perfective structure/marking (e.g., Jakobson, 1932, 1957, 1966; Forsyth, 1970; Dahl, 1985; Klein, 1994, 1995; Paslawska & von Stechow, 2003a; Borik, 2006; Willim, 2006; Kagan, 2008, 2010; Frackowiak, 2015; Klimek-Jankowska et al., 2018; Klimek-Jankowska & Błaszczak, 2021; Kwapiszewski, 2021).

The view of the imperfective aspect as an unmarked aspect value goes back at least to Jakobson (1932, 1957, 1966). In his view, based primarily on Russian, the imperfective aspect is an unmarked value of aspect, i.e., the absence of aspect specification – only perfective predicates are marked, i.e., specified for aspect. This basic idea has been adopted and elaborated by various scholars in both traditional (e.g., Forsyth, 1970) and formal implementations (e.g., Paslawska & von Stechow, 2003a; Borik, 2006; Kwapiszewski, 2021).

The notion of neutral aspect, which is usually attributed to Smith (1997), stands for compatibility with any kind of relation between the reference time and event time, and is originally proposed to apply only to grammatical systems that lack overt viewpoint aspect markers (see also Dahl, 2010, 88). Although Slavic imperfectivity is sometimes characterised as a notion close in spirit to that of neutral aspect (e.g., Klein, 1995), Smith herself analyses Russian imperfective verbs as instances of the imperfective aspect, due to their overt marking (either by unprefixed stems or by secondary imperfectivisers), modeling the imperfective semantics in terms of focusing on a part of a situation which includes neither the initial nor the final endpoint (Smith, 1997, 231). She considers Russian imperfective a parametric variant of the Universal Grammar Imperfective (*idem*: 234). Smith does follow Jakobson (1932, 1957) in assuming that the imperfective is unmarked, whereas the perfective is a marked value of aspect, but argues for the relevance of this opposition at the level of pragmatics, rather than semantics. Namely, as an unmarked form, the imperfective can be used to describe completed events (e.g., in the case of general-factual readings), but such readings arise only pragmatically, and do not pose a problem for a general definition of the imperfective aspect as entailing only a part of situation. There are also approaches that employ the neutral aspect as a separate imperfective operator (hence, defined positively) both in Slavic (Pancheva, 2003, 283-284, Iatridou et al., 2003, 171-174) and cross-linguistically (Schilder, 1995; Csirmaz, 2003; Dahl, 2010).

The term default aspect is due to Bohnemeyer and Swift (2004) and is employed in approaches that derive both aspectual values (i.e., imperfective and perfective) by means of a single zero operator that applies obligatorily at a certain point in the verbal derivation (cf. Mueller-Reichau, 2020, 91). Since default aspect makes use of a zero aspectual operator, in the majority of approaches applying it to Slavic languages, this means that traditional aspectual markers (i.e., prefixes and secondary imperfectivisers) are not seen as markers of grammatical aspect (e.g., Bohnemeyer & Swift, 2004; Mueller-Reichau, 2020; Biskup, 2023b,a), although in some approaches advocating default aspect secondary imperfectivisers are analysed as (exponents of) the (imperfective) aspectual head (Ramchand, 2004, 2008; Kwapiszewski, 2021), as elaborated in Sects. 2.2.1 and 2.2.2 below.

Having overviewed the different approaches to telicity and grammatical aspect, we can now return to the central topic of our paper – the syntax and semantics of secondary imperfectivisers. We first present approaches that analyse secondary imperfectivisers as markers of grammatical aspect (Section 2.2.1), after which we proceed to implementations divorcing secondary imperfectivisers from grammatical aspect (Section 2.2.2). Section 2.2.3 briefly overviews some recent approaches according to which secondary imperfectivisers spell out several heads rather than a single syntactic/semantic unit.

### Previous approaches to secondary imperfectivisation

#### Secondary imperfectivisers as markers of grammatical aspect

According to the most widespread view, secondary imperfectivisers are markers of grammatical aspect, more precisely: of the imperfective aspect (see Borik, 2006; Tatevosov, 2018 for a critical overview of Russian aspectual tradition, and e.g., Stevanović, 1989; Stanojčić & Popović, 1992 for the very similar Serbo-Croatian tradition). In formal syntactic and/or semantic approaches endorsing this idea, secondary imperfectivisers are analysed as aspectual heads and/or operators (e.g., Smith, 1997; Filip, 2000, 2005; Ramchand, 2004, 2008; Borer, 2005; Progovac, 2005; Borik, 2006, among many others).

For Smith (1997), secondary imperfectivisers are markers of the imperfective aspect, and consequently, secondarily imperfectivised verbs, just as simple imperfective stems, encode a part of a situation that does not include initial or final endpoints (Smith, 1997, 231). In Borik (2006)’s approach, secondary imperfectivisers are markers of imperfective viewpoint, which basically amounts to signaling that a verbal predicate is not perfective (see Section 2.1 above for Borik’s definitions of imperfective and perfective viewpoints). Filip (2000, 2005), while generally arguing that the Slavic verb and its morphology should be divorced from grammatical aspect, which is licensed by (covert) aspectual operators, considers secondary imperfectivisers the only morphemes that have a constant and purely aspectual meaning in all their occurrences (Filip, 2005, 145). As Filip puts it, this is possible if we assume that secondary imperfectivisers are inflectional, unlike prefixes, which are derivational morphemes. Tatevosov (2015, 2018) points out several drawbacks of such a proposal: e.g., some prefixes attach below, and some above secondary imperfectivisers (the former are usually referred to as lexical and the latter as superlexical prefixes), which would mean that derivational morphology with the same (aspectual) effect attaches both below and above an inflected verb form. Further, such an approach is forced to assume an additional, higher (perfective) aspectual layer above the imperfective layer, in order to capture the contribution of prefixes higher than secondary imperfectivisers. In later work, Filip herself abandons the analysis of secondary imperfectivisers as markers of imperfective aspect, arguing that the distinction between Slavic perfective and imperfective verbs (the latter subsuming secondary imperfectives) is to be understood as a lexicalised distinction operating at the level of the lexical verb (Filip, 2017, 185). Building on Filip (2008)’s notion of event maximisation and more generally Filip’s work on aspectual operators as partitivisers (e.g., Filip, 1993), Altshuler (2013, 2014) analyses the secondary imperfectiviser *yva* in Russian as an imperfective suffix, i.e., the imperfective partitive operator. More generally, he analyses both imperfective and perfective operators in Russian as aspectual partitive operators. Both of them are compatible with describing the event’s culmination, i.e., they both can express telic predicates. The difference is that the perfective operator requires proper event stages in the denotation of the *v*P it combines with, i.e., it requires a maximal stage (roughly, it combines only with bounded predicates), while the imperfective operator only requires a stage of an event in the extension of the VP it combines with. Crucially, this stage can be, but need not be maximal. According to Altshuler, the fact that the imperfective aspect is compatible with culminated events comes to the fore especially in the case of achievements, which are necessarily telic. The main difference between Altshuler’s and Filip’s approaches, on the one hand, and Smith’s approach, on the other, is that Altshuler’s and Filip’s imperfective operators semantically include any stage/part of the situation. In contrast, in Smith’s approach, initial and final parts/stages are excluded (with the fact that the imperfective can express completed events attributed to pragmatics).

According to Bohnemeyer and Swift (2004), there is only one (default) aspectual operator in Russian, which is zero-marked and its value is determined based on telicity properties. Telic predicates by default lead to the perfective viewpoint realisation of the aspectual operator, while atelic predicates without overt marking (i.e., traditional simple/primary imperfectives) are compatible with both imperfective and perfective viewpoints. However, due to pragmatic competition, structures with atelic predicates are interpreted imperfectively. Namely, the imperfective and the perfective interpretation constitute an entailment scale: since a telic predicate, which must be interpreted as perfective, is not used, it is inferred (due to the Gricean maxim of quantity) that the imperfective interpretation is intended (Bohnemeyer & Swift, 2004, 264-265). In their system, the secondary imperfectiviser marks imperfectivity ‘directly’, i.e., licenses the realisation of default aspectual operator as imperfective. Since in their approach imperfective viewpoint is based on the proper inclusion of the reference time into the event time, a problem arises as to how to account for those cases where (secondary) imperfectives are compatible with the opposite relation, where the event time is included in the reference time (e.g., in the so-called general-factual uses of imperfectives). This problem is avoided in Filip’s and Altshuler’s approaches discussed above by allowing the compatibility of the imperfective operator with any part/stage of the situation (see also Kwapiszewski, 2021’s and Mueller-Reichau, 2020’s approaches reviewed below, which are default-aspect approaches adjusted to solve this conundrum).

Ramchand (2004) takes secondary imperfectivisers in Russian to be generated outside the first-phase (*v*P) domain, based on the fact that they do not change the lexical meaning of the root nor the verb’s argument structure. In her approach, in the case of simple imperfective verbs, the aspectual head is by default realised by a null head, i.e., the t(ime) variable triggering an indefinite reference time interpretation. Prefixes, on the other hand, induce definite reference times (in the absence of secondary imperfectivisation). On this view, the secondary imperfectiviser is an overt exponent of the imperfective aspect, which also triggers an indefinite reference time. Since the secondary imperfectiviser combines with a *v*P containing a (lexical) prefix, which by hypothesis triggers a definite reference time, it has to take an event with presupposed time moments and somehow prevent it from being chosen as a (definite/specific) reference time. Ramchand proposes that the secondary imperfectiviser performs this task by introducing an ancillary event *e’*, whose time trace is free of presuppositions associated with the temporal trace of the event *τ*(*e*), making the time variable (= the Asp head) indefinite once again (for technical details, see Ramchand, 2004, 354-357). A similar claim is made in Ramchand (2008, 1704): the *(iv)aj* suffix in Russian is an aspectual head that asserts the existence of a subevent in the denotation of *v*P which is a process and which the time variable is linked to. The secondary imperfectiviser applies only to verbs that are subeventally complex (i.e., include the result phrase), but not to simple verbal stems, which only denote processes and not results. In addition, by economy, the aspectual head lexicalised by the secondary imperfectiviser cannot be merged unless required by some element higher in the structure, or by an interpretive effect.

In an approach coached within the framework of Distributed Morphology, Kwapiszewski (2021) analyses the imperfective aspect in Polish as the default interpretation of the unvalued aspectual head. On this approach, secondary imperfectivisers are also dissociated from the imperfective aspect – they are specified for the aspectual category without a value. It makes secondary imperfectivisers compatible with both perfective and imperfective interpretations, and, consequently, nothing prevents their combination with superlexical prefixes (which merge in the Spec,AspP, following common assumptions). More specifically, the aspectual head enters the derivation bearing an interpretable but unvalued aspectual feature [iasp:_]. Lexical prefixes bear an uninterpretable perfective aspect feature [uasp: pf] and move to the AspP to value [iasp:_] via Agree (*à la* Svenonius, 2004a). In this system, according to Kwapiszewski (2021), the simplest way to generate secondary imperfectives in the syntax/semantics is to make prefix movement optional. When the prefix stays in situ, the unvalued aspectual head is interpreted as imperfective, just like in the case of simple/bare imperfective verbs. Kwapiszewski further argues that aspect is realised by secondary imperfectivisers in Polish whenever a prefix is inserted at PF. He proposes that the verbal and aspectual heads (*v* and Asp) undergo Fusion as a default rule of Polish morphology, and hence a single terminal node [*v* Asp] results for lexicalisation, which is usually realised by the exponent of the verbal head (i.e., the theme vowel and/or verbalising suffix), in accordance with the Subset Principle. In the case of secondary imperfectivisation, the prefix hierarchically intervenes at PF between *v* and Asp, blocking Fusion. As a result, *v* and Asp are realised by two distinct Vocabulary Items, i.e., *v* by a verbalising suffix, and Asp by a secondary imperfectiviser, *-ow* and *-yw*, respectively. As the insertion of the secondary imperfectiviser depends on the PF phenomena, Kwapiszewski’s approach differs from other approaches presented in this subsection in that secondary imperfectivisation is primarily a *morphological* operation, rather than a syntactic or semantic one (see also Kwapiszewski, 2022).

Ramchand (2004, 2008)’s and Kwapiszewski (2021)’s analyses exemplify the approaches advocating default aspect introduced in Section 2.1, with only one aspectual operator and/or head, which receives its value depending on the structure it combines with. In their approaches, the secondary imperfectiviser is an overt exponent of this operator under specific syntactic, semantic or morphological conditions. In Section 2.2.2, we review some other default aspect approaches that do not take the secondary imperfectiviser as an exponent of the aspectual operator.

Finally, several prominent approaches argue for a radical split between simple imperfective verbs and secondarily imperfectivised verbs. Borer (2005) proposes a view according to which all affixed perfectives in Slavic are telic, basic (primary) imperfectives are atelic (i.e., unmarked for telicity), while secondary imperfectives crucially involve grammatical aspect. The fact that some prefixes scope over secondary imperfectivisers poses a problem for Borer: if all prefixes are generated in the inner aspect domain (telicity, quantity), as Borer argues, and secondary imperfectivisers are markers of grammatical aspect, prefixes scoping over secondary imperfectivisers should be impossible – contrary to the empirical facts. According to Progovac (2005), secondary imperfectivisers in Serbo-Croatian are generated in the projection hosting grammatical/outer aspect (the higher of two aspectual projections in her approach). Specifically, secondary imperfectivisers come with a quantificational feature of universal quantification comparable to *every/each*: just like these quantifiers scope only over count nouns, secondary imperfectivisers scope over quantifiable (perfective) events. Progovac (2005, 106) states that the universal quantification over “completed actions and times” can be accounted for straightforwardly if secondary imperfectivisers merge in the domain of grammatical aspect, assuming that Slavic perfectivity is generated in the domain of inner aspect. Progovac proposes that the semelfactive suffix -*nu* is also generated in the domain of grammatical aspect, coming with the feature of existential quantification, which ensures at least one event. Since these suffixes are generated in the same projection, according to Progovac, they are correctly predicted not to appear together, as in * (knock-suff-si-inf). While the complementary distribution of secondary imperfectivisers and the semelfactive suffix is used as an argument also in some other works (to be reviewed in 2.2.2), there are at least some verbs allowing the combination of these two affixes (Milosavljević, 2023b; Štarkl et al., 2024), which indicates that they are not generated in the same domain. Additionally, since prefixes for Progovac are markers of inner aspect (as in Borer, 2005), her approach also fails to account for the possibility of making secondary imperfectives perfective again by means of prefixation.

#### Secondary imperfectivisers below grammatical aspect

In this subsection, we present approaches that divorce secondary imperfectivisers from grammatical aspect. We will first review approaches in which traditional aspect morphology is separated from grammatical aspect, but both imperfective and perfective viewpoints may be fully predicted based on traditional aspectual markers. We then turn to approaches in which the exact value of grammatical aspect is not fully predicted by the traditional aspect morphology.

In Klein (1995)’s view, based on Russian data, grammatical aspect depends on containment relations between the reference time and event time, and can thus only emerge in a finite clause, and not at the level of the verbal stem. He proposes that Slavic imperfective and perfective verbs are lexically and morphologically specified for being compatible with imperfective and perfective viewpoint aspect, respectively, but they do not express grammatical aspect by themselves. In Klein’s analysis, simple verbs express 1-state contents, which roughly correspond to atelic predicates, consisting of just a source state in his terminology. Prefixation results in a complex, 2-state content predicate, where the initial state is a source state, and the resulting state is labeled the target state. Finally, the secondary imperfectiviser added to a 2-state verbal predicate marks its source state as a distinguished state, i.e., the only of the two states that is relevant for the aspectual interpretation. In other words, secondary imperfectivisers do not ‘delete’ a target state from the lexical content of the verb, but indicate that only the source state is relevant for the aspectual interpretation, i.e., falls in the scope of reference time. For the perfective aspect to emerge, the reference time must have a common subinterval with both source and target states at the same time. Since this is only possible for 2-state predicates, 1-state verbs are automatically imperfective. To license imperfective aspect, the reference time must have a common subinterval only with a source state that functions as a ‘distinguished’ state, but not with a target state. This holds for 1-state predicates and for those 2-state predicates for which the source state is marked as distinguished, i.e., those expressed by secondary imperfectives. Since the reference time can overlap with a distinguished state in different ways (can be included in it, equal to it or even contain it), imperfective aspect has a very wide range of applications, i.e., it behaves as a neutral, unmarked aspect. In a nutshell, perfective aspect emerges when the reference time extends over both the source state and the target state, while imperfective aspect is licensed when the reference time only affects the distinguished state, which is the only state in 1-state expressions, and the source state in 2-state expressions.

Tatevosov (2015, 2018) proposes that the secondary imperfectiviser in Russian is generated inside the external-argument projection (i.e., inside the *v*P in his terminology, to be understood as the VoiceP). This proposal is part of his general agenda of separating Russian verbs from grammatical aspect, according to which, similar to Klein’s view, grammatical aspect emerges at the clausal level, where the verb is already fully derived and only some inflection features may be added. Tatevosov analyses Russian secondary imperfectiviser *yva* as an eventiser in the sense of Paslawska and von Stechow (2003b), i.e., it combines with a relation between events and states (of the type 〈 v, 〈 v, t 〉 〉), existentially binds the state and yields a property of events. Tatevosov supports this claim with the incompatibility of Russian secondary imperfectives with passive participles in Russian, which are not eventive. This is expected if the passive participle needs a semantically active state argument, which is already bound by the suffix *yva*. Although aspectual morphemes, including *yva*, do not express grammatical aspect, in Tatevosov’s approach, just as in Klein’s, both perfective and imperfective viewpoints can be predicted based on traditional aspectual morphology. He follows Klein (1995) in analysing perfective and imperfective operators as type-theoretically different: the perfective operator takes a relation between events and states (〈v, 〈v, t〉 〉) as its argument, whereas the imperfective operator takes a property of events (〈v, t〉). The first type of argument is brought about by (prefixed) perfective verbs, while the latter is expressed by imperfective verbs.

According to Mueller-Reichau (2020), verbal roots in Slavic denote eventuality kinds. Lexical prefixes modify eventuality kinds which are 1-state predicates, turning them into 2-state predicates (i.e., 〈source state, target state〉 predicates in the sense of Klein, 1994, 1995). The role of secondary imperfectivisers is to re-categorise the stem as belonging to the class of 1-state predicates, which they accomplish by making the target state truth-conditionally irrelevant, i.e., invisible for the default aspectual operator during the computation of grammatical aspect. Secondary imperfectivisers, just like lexical prefixes, apply within the lexical stage (i.e., Ramchand, 2004, 2008’s first phase). The default aspectual operator, which is zero-marked, is then sensitive to whether a predicate at the lexical stage expresses a 1-state predicate, resulting in the imperfective interpretation, or a 2-state predicate, yielding the perfective interpretation. Mueller-Reichau’s proposal is similar in spirit to Klein’s and Tatevosov’s in the sense that grammatical aspect is separated from aspectual morphology and depends on the relation between the source and target states. However, Mueller-Reichau’s proposal differs from Klein’s and Tatevosov’s in employing only one (default) aspectual operator: instead of separate perfective and imperfective operators, there is only one operator, which is zero by default and depends on the structure it combines with. In this latter sense, Mueller-Reichau’s approach is akin to the approaches of Bohnemeyer and Swift (2004), Ramchand (2004, 2008) and Kwapiszewski (2021), reviewed in the previous subsection, which also employ one (default) operator. The difference is that these works, unlike Mueller-Reichau (2020), consider the secondary imperfectiviser an (exponent of an) aspectual head, either imperfective (Bohnemeyer & Swift, 2004; Ramchand, 2004, 2008) or default (Kwapiszewski, 2021).

Biskup (2023b,a) also argues that grammatical aspect should be divorced from traditional aspectual morphology (in Czech and Russian). In his approach, there are multiple projections whose heads bear imperfective or perfective features, which are hierarchically ordered in the following way: . In prose, a perfective feature (pf) can be contributed by lexical prefixes (LPs) in the root phrase, by the semelfactive suffix (SEML) in the *v*P, or by two projections (SPPs) headed by superlexical prefixes (SLPs). The secondary imperfectiviser hosts its own projection (SIP) sandwiched between the two SPPs and is the only head that bears an imperfective feature (ipf). Biskup argues that the aspect value is determined via the aspectual head agreeing with the structurally highest aspectual marker: whenever some of the heads bearing a perfective feature is the structurally highest, the aspectual head gets perfective value; when the secondary imperfectiviser is the last piece of information available in the structure, grammatical aspect (morphological aspect in his terminology) is interpreted as imperfective. In the case of simple stems, grammatical aspect gets an imperfective interpretation by default. A similar approach is offered in Klimek-Jankowska and Błaszczak (2022, 2023), who also propose that secondary imperfectivisers are merged in their own projection between the *v*P and the grammatical aspect. They follow Tatevosov (2015)’s arguments that the grammatical aspect should be kept apart from traditional aspectual morphology. The motivation for the base-generated position of secondary imperfectivisers above the *v*P in their approach comes from idioms. The authors assume that the *v*P is the domain of the idiomatic interpretation, and since secondary imperfectivisers do not affect the idiomaticity of *v*P, they must be generated above it (as all items within the *v*P can affect its idiomaticity).

Łazorczyk (2010) considers that Slavic imperfective verbs and perfective verbs reflect atelicity vs. telicity rather than the distinction in grammatical aspect, which depends on the relationship between the event time and reference time. Based on the temporal interval test (introduced in Section 2.1), she concludes that Slavic imperfective verbs are compatible with both imperfective and perfective viewpoints, while perfective verbs are always embedded under the perfective viewpoint. This state of affairs is corroborated by the fact that in at least some Slavic languages (e.g., Bulgarian, Old Church Slavonic, Serbo-Croatian), the imperfective–perfective distinction is grammaticalised through the availability of verbal forms (aorist vs. imperfectum), which cut across traditional imperfective verbs and perfective verbs. Within such a system, the primary role of secondary imperfectivisation is to apply to a telic predicate and return an atelic/homogeneous one. More formally, the secondary imperfectiviser operator takes an event of which predicate *P* holds, and returns a homogeneous subpart of that event (*idem*: 138). The imperfective viewpoint, unlike the secondary imperfectiviser, does not make reference to the event’s internal structure, but only to its temporal duration. Once the secondary imperfectivisation has been applied, either of the two viewpoints (imperfective or perfective) can arise. Syntactically, the secondary imperfectiviser operator is generated as the head of the InnerAsp[SI] phrase, which merges on top of InnerAsp[telic] phrase (hosting prefixes) (*idem*: 139). In secondary imperfectives, the semantic contribution of the prefix remains in the form of the existence of some culmination point, which is not part of the asserted event but exists in the possible extension of the timeline on which the event is located (*idem*: 136). Superlexical prefixes that merge above secondary imperfectivisers are hosted in yet another telic projection above the InnerAsp[SI] (*idem*: 255). Łazorczyk (2010)’s approach, hence, employs three (a)telicity-related projections stacked on one another. This is similar in spirit to Biskup’s proposal in the sense that different types of prefixes and the secondary imperfectiviser head their own projections. However, Łazorczyk employs (a)telicity projections to host traditional aspectual markers, while for Biskup they all head their own (possibly qualitatively different) projections. Łazorczyk’s approach is different from all other approaches reviewed in this subsection in that the viewpoint is only partly predicted by (telicity) projections hosting prefixes and suffixes: a prefixed telic structure will necessarily end up embedded into the perfective viewpoint projection, while an atelic structure, including the one headed by secondary imperfectivisers, may be selected by both imperfective and perfective viewpoint structures. In contrast, for Klein (1995), Tatevosov (2015, 2018), Mueller-Reichau (2020), Biskup (2023b,a), the value of the viewpoint aspect is fully predictable by the last marker available in the structure: whenever it is a prefix or a semelfactive suffix, the perfective aspect emerges, otherwise it must be imperfective.

According to Markman (2008), the semelfactive suffix -*nu* and the secondary imperfectiviser -*iv* in Russian are both exponents of a single *v*P-selecting light verb v, which denotes an atelic event and is merged above the lexical prefixes. The light verb is realised as *-nu* in the presence of feature [+Instantaneous] and as a secondary imperfectiviser in the absence of other features, or in the presence of features [+Progressive]/[+Habitual]. The single-head approach to the two suffixes is based on the claim that they are in complementary distribution in Russian. Their status as light verbs is motivated by the similar behavior to light verbs cross-linguistically. For instance, just as light verbs in Yiddish, these two suffixes have an aspectual effect, can be productively added to a verbal stem (producing predictable meaning changes), both lack their own argument structure (i.e., do not change the argument structure of a lexical verb they combine with), cannot be used with non-eventive verbs (at least in Russian), etc. Relatedly, Armoškaitė and Sherkina-Lieber (2008) argue that the Russian secondary imperfectiviser -*yva* is a pluractional marker, i.e., the plural counterpart of the singularity (uniactionality) marker -*nu*, and the two suffixes occupy the same syntactic slot. This is evidenced, as in Markman (2008), by their complementary distribution in Russian. Armoškaitė and Sherkina-Lieber (2008) argue that the secondary imperfectiviser in Russian is not an aspectual suffix based on the following two additional arguments. First, in certain cases, -*yva* is added without any aspectual contribution, just to make the semantics of the stem more congruent with the semantics of the prefix (e.g., the cumulative *na*- in Russian does not combine with perfectives, so the secondary imperfectiviser is required as a ‘mediating’ step: while *na-da-t’ (na-give-inf) is not felicitous, *na-da-va-t’* (na-give–si-inf) is). And second, the suffix -*yva* can be added (in Russian) to at least some affixed imperfective verbs.

#### Multiple secondary imperfectivisers

While the above approaches focus on the secondary imperfectiviser as a syntactically and/or semantically unique morpheme, some recent approaches argue for multiple secondary imperfectivisers. For instance, based on the interaction between secondary imperfectivisation and the delimitative prefix *po-*, Biskup (2024) presents arguments for two structural positions of the secondary imperfectiviser in North Slavic (Russian, Polish, Czech): one below this prefix (with the progressive syntax and semantics) and one above it, which encodes plural semantics. Klimek-Jankowska and Simeonova (2024) compare Bulgarian to Polish and conclude that Bulgarian distinguishes two types of secondary imperfectives, while only one type is found in Polish. Their evidence comes from the fact that Bulgarian, unlike Polish, systematically allows “aspectual triplets”, where the secondary imperfectivising suffix can merge on top of a verb with a so-called purely perfectivising prefix, yielding exclusively an iterative interpretation (in contrast to the secondary imperfectivisation of verbs with lexical prefixes, where both progressive and iterative interpretations are possible). These authors follow Cinque (1999)’s Cartographic enterprise in assuming two aspectual positions for secondary imperfectivisers in Bulgarian – a higher one (Asp_habitual_) and a lower one (Asp_progressive_). As is clear already from this brief overview, argumentation for multiple positions and/or different semantics of secondary imperfectivisers comes from comparing different Slavic languages. For even more fine-grained comparisons including larger sets of Slavic languages, the reader is referred to Klimek-Jankowska et al. (2024) and Wiemer et al. (2025).

## Present proposal: secondary imperfectivisation is reverbalisation

Our approach to secondary imperfectivisers shares certain aspects with most of the views discussed in Section 2. Building on Jakobson (1932, 1957, 1966), Forsyth (1970), Dahl (1985), Klein (1994, 1995), Paslawska and von Stechow (2003a), Borik (2006), Willim (2006), Kagan (2008, 2010), Frackowiak (2015), Klimek-Jankowska et al. (2018), Klimek-Jankowska and Błaszczak (2021), Kwapiszewski (2021), Arsenijević (2023), Milosavljević (2023a,b), we take traditional imperfective verbs to be unspecified for aspect, with perfectives being marked and restricted to a particular interpretation. Like Klein (1995), Łazorczyk (2010), Tatevosov (2015, 2018), Mueller-Reichau (2020), a.o., we sever grammatical aspect from the lexical verb, i.e., from the structure below tense and person. Similarly to Bohnemeyer and Swift (2004), Mueller-Reichau (2020), and in full agreement with Łazorczyk (2010), Arsenijević (2023), Milosavljević (2023a,b), we set the locus of difference between the two verbal classes at the level of inner aspect. In combination, these three components yield a view in which the binary opposition traditionally labeled in terms of perfectivity is one concerning telicity, where traditional perfective verbs are marked and restricted to the telic interpretation, while traditional imperfectives are unspecified, thus aspectually unrestricted. Their tendency for atelic interpretations emerges pragmatically from a scalar relation of strength (Horn, 1989), i.e., from scalar implicature (Heim, 1991).

The crucial observation for our analysis is that under the standard definitions, atelic predicates are actually entailed by their telic minimal pairs, i.e., that the extension of a telic predicate is included in the extension of its minimal atelic counterpart.

Krifka (1998), Borer (2005), Arsenijević (2006), a.o., model telicity in mereological terms as quantisation, i.e., lack of cumulativity and divisiveness. Take a simple *v*P, such as *drink a glass of milk*. As pointed out by Krifka (1998), telicity is a property of predicates (i.e., ways of describing eventualities, and referring to them), and not of eventualities themselves. Eventualities, for instance of drinking a glass of milk, can be described by telic as well as atelic predicates, as illustrated in (4). Temporal viewpoint semantics aside, the telic verbal predicate in (4a) may refer only to a complete event of drinking a glass of milk. If John drank from a glass of milk, but did not finish it, (4a) is false. The atelic verbal predicate in (4b) refers to a set of events that include both complete and incomplete events of drinking a glass of milk, as well as sums thereof. Hence, (4b) is true for both of the above situations, that where John has finished the glass (or any other intended amount), and that in which he has not.

(4)
  - John drank a glass of milk in/??for an hour.
  - John drank milk ??in/for an hour.

The opposition between telicity and atelicity can be best understood as follows: a telic predicate is an intersection of an atelic predicate and a restriction to complete events in some explicitly or contextually specified sense. In line with Borer (2005), Łazorczyk (2010), Arsenijević (2006, 2023), Milosavljević (2023a,b), we propose a minimal model for this fact: a bare verb, the *v*P, is unspecified for aspect, and telicity emerges by the projection of the respective phrase on top of *v*P, one headed by the feature that introduces the relevant restriction, which we label QP. More specifically, we assume the view of inner aspect from Milosavljević (2023b) and Arsenijević (2023), whereby atelic verbs (traditional imperfectives), i.e., verbal expressions headed by them, denote predicates unspecified for both units of counting and number. Observe another example: the expression  ‘read^IPFV^ War and Peace’ denotes the set of sums of parts of events of reading the novel (thus also the middle 4 hours of John’s reading of the novel last month, but also the sum of Mary’s reading of War and Peace, Bill’s reading of the first 10 pages, and John’s reading it three times, as well as just entire reading by John). The verb  ‘sleep^IPFV^’ denotes any John’s sleeping 2 hours, as well as the sum of the first half of his sleep and Mary’s sleep for 20 minutes. Telic verbs (traditional perfectives) denote predicates whose denotation includes only singular atomic events. For instance, the denotation of  ‘read^PFV^ War and Peace’ includes the full event of John reading the novel, the full event of Mary reading the novel, the full event of Bill reading the novel, but not any proper part of any of these, or any sum thereof that is smaller or larger than one individual event of reading the entire book. The denotation of the expression  ‘sleep^PFV^’ includes only single individual events bounded by a contextually provided restriction of John, Bill and Mary (considering that they constitute the domain of individuals) sleeping. Telic verbs are in this view more restricted than atelic verbs, as they impose the restriction of reference to single atoms. Secondary imperfectivisation can be seen as a removal of these restrictions, i.e., as reverting back to the unconstrained interpretation, and our approach is more in line with the latter metaphor. More formally, the new *v*-head is a verbal universal grinder (Pelletier, 1991; Rothstein, 2017): it maps the set of atomic events constituting the denotation of the selected telic predicate onto a set of proper parts of those events and all their possible sums. The syntactic difference between aspectually unspecified and telic predicates is illustrated in (5).^9^

(5)

In English, atelic and telic meanings are not morphologically marked, but can be induced from the interaction between the atomicity of a given predicate and the specification of viewpoint aspect and tense. For instance, in (4a), the simple past tense combined with an atomised predicate induces a telic interpretation, while in (4b), the same form in combination with a non-atomised predicate yields atelicity. In Serbo-Croatian, however, as well as in other Slavic languages, telicity is marked by a prefix or the suffix *-nV* (Borer, 2005; Łazorczyk, 2010; Arsenijević, 2023).^10^ A verb without such an affix with an atomised meaning is unspecified, and projects a verbal expression whose extension includes both the set of individual whole atoms and the set of their parts, as well as all sums formed within the union of these two sets.

We argue that it is a principled property of syntax that a semantic restriction introduced by a functional projection can restrict the set of possible higher projections (by being incompatible with some of them), and can be further restricted by those that are compatible with it, but crucially cannot be undone within the same extended projection. In the particular case of telicity, atelic, i.e., unspecified predicates, allow for all the possible kinds and values of the higher projections licensed by the category *v*, which then further restrict the meaning of the *v*P. A telic predicate, i.e., a predicate denoted by a verb that does include an affix marking telicity (i.e., a QP), is restricted to telic interpretations, and incompatible with some particular (specifications of) higher heads within the same extended projection. Projections that it is compatible with further restrict its interpretation, but they cannot undo the restriction to telicity. Our model thus makes the following prediction. Atelic Serbo-Croatian verbs, on the one hand, should be compatible with all verb forms and contexts, but should on pragmatic grounds tend to lose competition with their telic counterparts wherever such counterparts are available and compatible with the context, since in such cases the latter are more specific expressions of the same meaning. Telic verbs, on the other hand, should be compatible only with certain verbal forms and contexts, in which they should be preferred to their atelic minimal pairs. As shown by Arsenijević (2018), this is exactly the case.

This view has already been implicit in the literature, especially on the compositional derivation of verbal aspect. E.g., Borik (2002, 43) uses contexts that exclude perfective verbs (our telic verbs) to verify that a verb is imperfective (i.e., atelic), but not contexts that license them to verify that a verb is perfective (in our view, telic). Similarly, Łazorczyk (2010) shows that telic verbs necessarily include completion, but does not show that atelic verbs necessarily exclude it. In Serbo-Croatian, as predicted, imperfective viewpoint aspect verb forms, such as the imperfectum in (6a) or the present participle in (6b), as well as imperfective viewpoint contexts such as parallel duration adverbial clauses as in (6c)^11^ are incompatible with telic verbs. Perfective viewpoint aspect verb forms, such as the aorist in (6d) (Arsenijević, 2013b) and the past participle in (6e), and perfective viewpoint environments, such as temporal posteriority adverbial clauses in (6f), are compatible with both telic and atelic verbs, but prefer the former.^12^

(6)
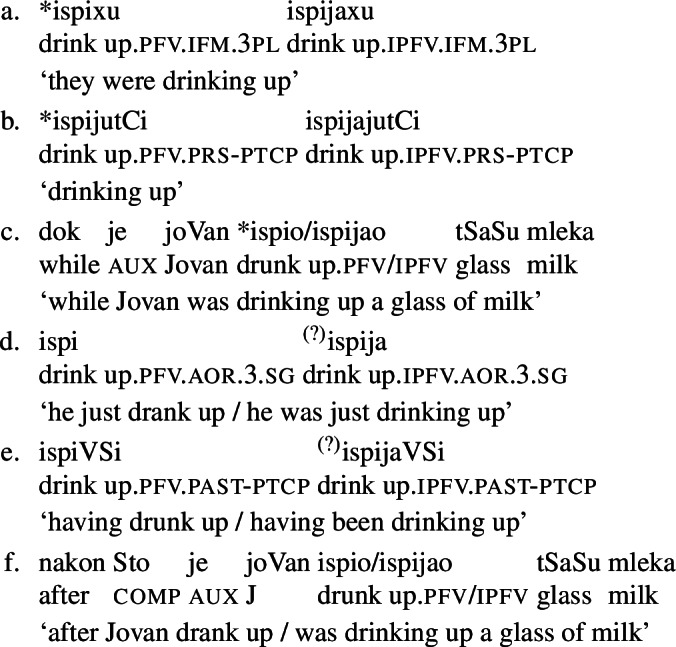

Affixes marking telicity are often not semantically exhausted by this marking, but contribute additional semantics. For illustration, consider (7), where after prefixation, the verb changes meaning from ‘watch’ to ‘examine’ and from ‘demolish’ to ‘disguise’, and after suffixation mainly to a minimal instance meaning, i.e., ‘throw a quick look’ and ‘do a minimal instance of demolishing’. With prefixes, additional semantics is the default case, and in fewer cases the contribution is purely aspectual, while with the suffix , the opposite is the case, i.e., the contribution of the suffix is, more often than not, purely aspectual. When a telicising affix brings about additional meaning, the derived verb may typically undergo further imperfectivisation, i.e., reverbalisation in terms of our analysis.

(7)
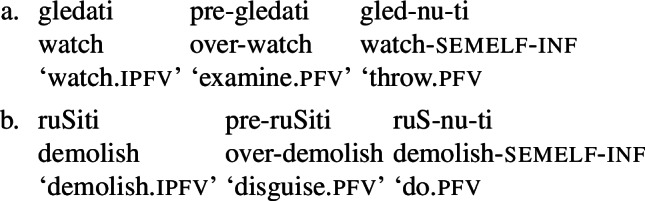

The derived meanings too need to have an atelic way of expression. The base verb lacks the lexical semantics added by the telicising affix, and once the meaning of the verb is restricted to telicity, no further restriction can revert it to the atelic interpretation. On our analysis, Serbo-Croatian overcomes this problem by deriving a new verb from the telic one (more precisely from the respective QP). This is done by merging the QP with a *v-*head, thus deriving a new *v*P, i.e., a new lexical verb. The specific *v*-head that is needed for this needs to carry a selection restriction to verbal bases and to impose the verbal category to the derived structure. It is hence best represented as a pair of an uninterpretable and an interpretable [v] feature, the former as a selectional restriction and the latter as the categoriser. As the vocabulary items realising the feature [v] are theme vowels, reverbalising *v*-heads are realised as a sequence of two theme vowels. Together with the theme vowel of the base verb, this gives the maximal sequence of three theme vowels, which is attested in the morphophonological analysis. This is schematically represented in (8).^13^

(8)

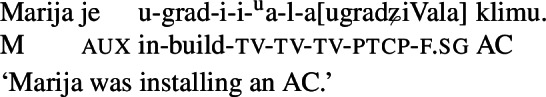

The new *v*P then projects its own extended projection without a (specified) QP, thus deriving an aspectually unspecified interpretation, fitting the traditional notion of imperfective verbs.

In this paper, we do not discuss the issue of recursive reverbalisation in detail, but we point out that in at least some Slavic languages, Serbo-Croatian included, there seem to hold a morphophonological restriction on the repetition of the same morphemes (see, e.g., Menn & MacWhinney, 1984 for The Repeated Morph Constraint). Žaucer (2009) also claims that multiple secondary imperfectivisation (reverbalisation in our terms) in Slovenian is possible, but the suffix can be morpho-phonologically realised only once (see also Section 3.1.2 for the possibility of null reverbalisers). While a detailed empirical overview would go beyond the scope of this article, we observe that in some cases re-reverbalisation is relatively acceptable. As expected, these are the cases where the two reverbalisers are exponed by rather different surface patterns. For instance, in the example (9a), the first round of reverbalisation is realised by a change on the root ( becomes ) and a theme vowel. The second reverbaliser is realised as . While the outcome is not fully acceptable, given that it contains a repetition of the same morpheme, the surface dissimilarity makes the result marginally acceptable. On the other hand, the result of re-reverbalisation in (9b), where both reverbalisers are exponed as *ava*, is fully unacceptable.

(9)
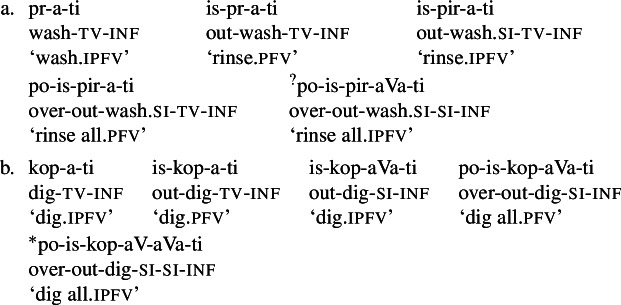

The very option of reverbalisation is restricted by the nature of perfectivisation that precedes it. If the perfectivisation took place without adding lexical meaning, reverbalisation is elsewhere-blocked by the fact that the reverbalised verb would have exactly the same meaning as the original imperfective verb that got perfectivised. If the perfectivisation came with a lexical semantic enrichment, reverbalisation is possible, as the derived meaning needs an atelic way of expression. This is illustrated in (10), where  and , which are pure telic counterparts of  ‘write’ and  ‘paint’, respectively, cannot be reverbalised, while  ‘write out’ and  ‘repaint’, which have additional lexical semantics, readily reverbalise.

(10)
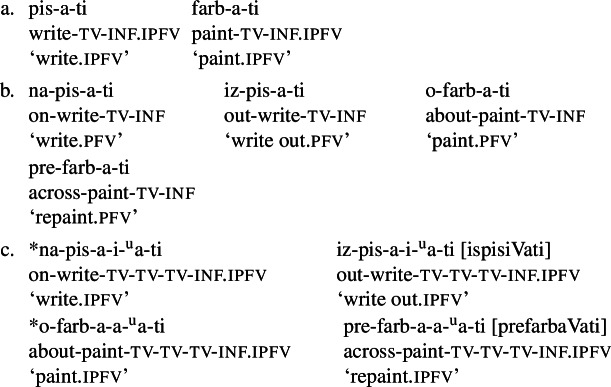

Except for verbs with purely perfectivising prefixes, some verbs with quantificational prefixes also resist reverbalisation. The typical example in Serbo-Croatian is the delimitative prefix  in verbs such as  ‘lie down (for about twenty minutes)’. Here again, the reason is arguably the fact that the imperfective version with the same meaning can be expressed by the base verb:  ‘lie down (for about twenty minutes)’; see Milosavljević (2022) for an analysis of this prefix in Serbo-Croatian. Other cases where secondary imperfectivisation is not possible include verbs that already have a complex categorising affix, i.e., those that are already reverbalised, as discussed above.^14^

The proposed model, in which the meaning of atelic verbs subsumes telicity and their non-telic interpretation is a consequence of scalar implicature or pragmatic strengthening, correctly predicts the existence of three classes of atelic verbs. The first are those which are fully ambiguous between telic and non-telic interpretations, and which emerge when there is no telic minimal pair against which they would lose competition in telic contexts. These are the so-called biaspectual verbs (11a). The second class displays an inclination towards non-telic interpretations, emerging from competition with a derived, and hence more marked telic counterpart (11b). The third are those cases in which the atelic verb derives from the telic counterpart via secondary imperfectivisation, i.e., reverbalisation (11c). In this case, the non-telic pragmatic preference is even stronger because the aspectually unspecified verb is not only less specific in telic contexts, but also more costly due to higher morphological complexity.

(11)
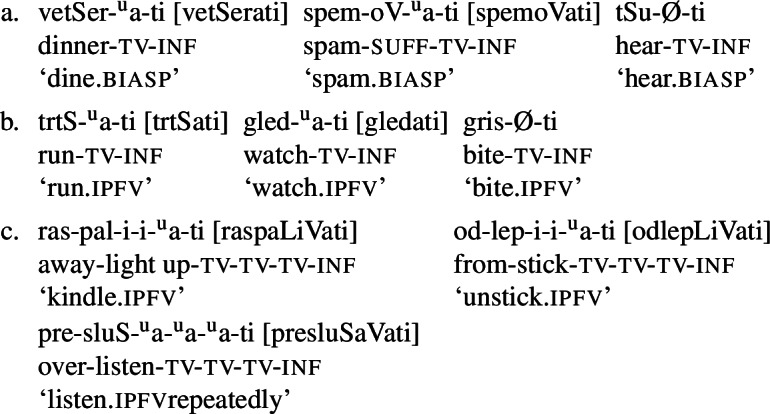

The analysis of the non-telic tendency in terms of competition predicts that it can be cancelled. This prediction too is borne out. Consider the dialogue in (12). The question licenses both an unspecified interpretation, ambiguous between the telic and the atelic reading, to which the fitting answer is affirmative (B interprets the verb as ambiguous and picks up the telic subset of interpretations), and a strictly non-telic one, on which the cooking will not be completed on the following day, and which fits the negative answer (B interprets the verb as strictly non-telic, and puts focus on the telic marking on the verb in order to cancel the strict non-telic interpretation).

(12)
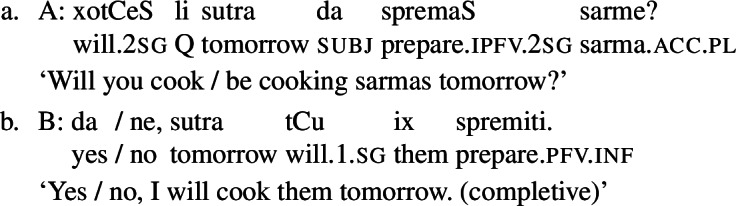

Secondary imperfectivisation is thus better analysed as secondary verbalisation. Bare *v*Ps are atelic, which, we argue, means that their extensions include events which satisfy telicity requirements on a par with those that do not. They may be restricted to telicity, and simultaneously also semantically enriched, by the projection of a QP. Once introduced – the telicity restriction cannot be removed within the same extended projection. The only way to revert back to aspectual unspecifiedness is to derive a new verb by merging in a new verbal head, which then projects its own extended projection without telicity, and establishes atelic, i.e., aspectually unspecified reference. Unless, of course, it is telicised again, if this is licensed by added lexical semantics.^15^

### Extensions and residual issues

So far, we have been mostly concerned with typical patterns where the derivation chain includes a simple imperfective verb, which can get perfecivised, and then revebalised (and again perfectivised). In this section, we extend our analysis to some minor patterns: the cases with simple perfective verbs that also undergo secondary imperfectivisation (Section 3.1.1), and cases of perfective verbs with multiple prefixes but without (overt) reverbalisation (Section 3.1.2).

#### Simple perfective verbs and secondary reverbalisation

Serbo-Croatian, like other Slavic languages, disposes of a small, closed class of simple perfective verbs, that is, verbs that consist of just a root, a theme vowel, and an inflectional ending but are perfective rather than imperfective. These verbs, like prefixed perfective verbs, can undergo secondary imperfectivisation, i.e., reverbalisation, as illustrated in (13). Some such verbs take default reverbalisers ( in (13a-13b)), while in other cases less productive reverbalisers may occur, such as (descriptively)  and . While  is completely idiosyncratic as a reverbaliser,^16^ all other patterns, both productive and unproductive, are shared with prefixed perfective verbs (see Simonović et al., 2023, who also offer a morpho-phonological analysis of less productive patterns in terms of the sequence of theme vowels).

(13)
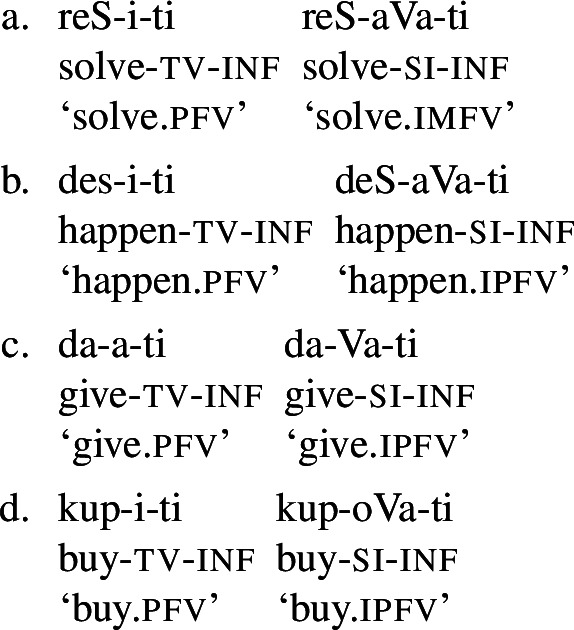

One analytical possibility for the patterns in (13) is to treat simple perfective verbs as prefixed by a null prefix, which licenses telicity, just as ‘regular’ prefixes as proposed in Borer (2005), Łazorczyk (2010), or Milosavljević (2023b), and then the reverbalisation applies. Although the null prefix does not contribute lexical meaning, the secondary imperfective is motivated exactly because there is no “primary imperfective”, so the relevant aspectual counterpart must be derived by suffixation, i.e., by the sequence of theme vowels on our analysis. In this sense, what descriptively looks like an instance of prefixation of an already perfective verb, such as  (apart-solve) ‘(re)solve’, is actually a regular procedure where the verb is marked as telic due to prefixation (here the prefix  has the same effect as the null prefix in  ‘solve’). The question remains, then, why there is no simple imperfective verb  ‘solve’. We submit that this is an idiosyncratic pattern similar to what can be observed also with ‘regular’ prefixes. Namely, there are verbs with overt prefixes as well that lack simple imperfective counterparts, such as  ‘begin’, which is not attested in the contemporary Serbo-Croatian without a prefix (*), although the same root/stem appears with various other prefixes as well (see also Borer, 2005 for further discussion of such gaps in Slavic languages).

Alternatively, one can treat simple perfective verbs as listed verbs with perfective semantics (Arsenijević, 2023), as there are few such verbs and the class is not productive anymore. Specifically, in the database WeSoSlaV (Arsenijević et al., 2024), which comprises 5.300 most frequent Serbo-Croatian verbs, they constitute 4.12% of simple verbs and 0.53% of all verbs. Since WeSoSlaV contains the most frequent verbs, which tend to be more idiosyncratic, this percentage is probably even lower when one considers low-frequency verbs or loanverbs. We have not been able to identify any new simple verb (e.g., adapted loan verb) that is purely perfective.

#### Multiple prefixation without (overt) reverbalisation

In Serbo-Croatian (as in other Slavic languages), it is also possible to have multiple prefixation without (overt) secondary imperfectivisation/reverbalisation as an intermediate step. Such cases are illustrated in (14). The first example (14a) includes a typical derivational chain (simple imperfective – prefixed perfective – secondary/reverbalised imperfective – perfective with a quantification prefix), with the addition that another quantificational prefix is merged on top of that structure (the superlexical prefix  adds exhaustivity, while  contributes a distributive interpretation). The chain in (14b) is a less typical pattern: it includes what is usually regarded as simple perfective, and then two prefixes on top without any ‘imperfectivising’ step.

(14)
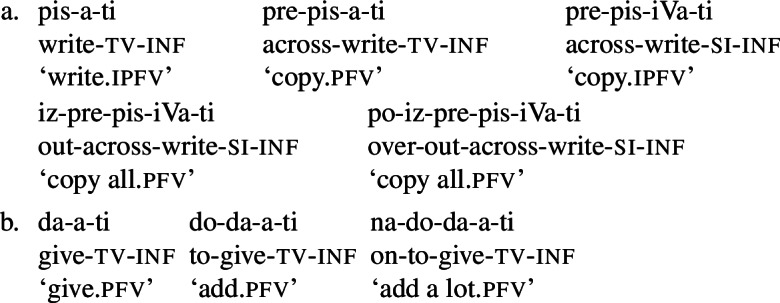

A possible way to account for these patterns, which is fully compatible with our current proposal, is that such verbs get reverbalised, but with a null verbaliser, as discussed in Milosavljević (2023b, 190-191). This idea is built on Žaucer’s (2009, 2010) proposal that such verbs might include a null light verb (which would correspond to *v* in our approach). An argument for such an analysis comes from the fact that for some speakers of Serbo-Croatian, certain verbs with multiple prefixes are available both without an (overt) reverbaliser (or tentatively, with a null one) and with a verbaliser (traditionally, secondary imperfectiviser). For instance, the verb  ‘add a lot’ from (14b) includes two prefixes on top of the verbal stem, but there is also a synonymous perfective verb  (on-to-give-si-inf) that has an additional, overt secondary imperfectivising suffix that syntactically and semantically ‘mediates’ between the lower  and the higher prefix . For at least some speakers of Serbo-Croatian, and especially in South-East Serbo-Croatian, the example in (14a) with multiple prefixes can also appear without the secondary imperfectiviser .

## Conclusion

We have argued for an analysis of secondary imperfectivisation in Serbo-Croatian as reverbalisation, i.e., an operation that takes a perfective verb, and turns it into a bare *v*P. On the morphophonological side, in Serbo-Croatian reverbalisation amounts to stacking theme vowels (see Simonović et al., 2023), which have independently been argued for to act as verbalisers (see Milosavljević & Arsenijević, 2022 for Serbo-Croatian). In this way, the inner aspect of the verb is reset to the unvalued, i.e., aspectually unrestricted default. The newly derived verb gets interpreted as imperfective by scalar implicature: were the perfective interpretation intended, the speaker would have used the more specific and computationally cheaper expression involving the perfective verb. The paper offers a simpler analysis of ‘imperfectivising’ suffixes in terms of theme vowels, i.e., it unifies ‘imperfectivising’ suffixes and theme vowels both morphologically and syntactically. This also means that simple and derived imperfective verbs receive a unified account – they are all bare *v*Ps, unspecified for telicity and grammatical aspect.

While our proposal builds on the literature on Slavic aspect, our empirical basis was restricted to Serbo-Croatian. Perhaps the most important issue that further research will have to address is therefore to what extent our analysis applies to Slavic secondary imperfectivisation in general. When evaluating this, it is important to distinguish between the general mechanism – reverbalisation – and its specific implementation in Serbo-Croatian, namely the stacking of theme vowels. Working on the most closely related language to Serbo-Croatian, Slovenian, Simonović and Mišmaš (2023) argue that while some secondary imperfectivisers can be analysed as theme vowel material, the most frequent secondary imperfectiviser, *ova*, needs to be analysed as containing the light multifunctional element *ov*, which also shows up in many other environments, plus a theme vowel. This may be a different mechanism of reverbalisation, whereby semantically vacuous lexical material is added. It is, of course, an empirical question whether the same could be applied to secondary imperfectivisers in other Slavic languages, such as Russian *yva* and Ukrainian *uva*.

## Abbreviations

acc: accusative
aor: aorist
aux: auxiliary
biasp: biaspectual
comp: complementiser
f: feminine
ifm: imperfectum
inf: infinitive
ipfv: imperfective
m: masculine
pl: plural
prs: present
ptcp: participle
semelf: semelfactive
sg: singular
si: secondary imperfectiviser
subj: subjunctive
suff: suffix
tv: theme vowel
pfv: perfective

## Footnotes

1 As discussed in more detail in Section 3, not all perfective verbs undergo secondary imperfectivisation. Slavic languages also differ with regard to which types of perfective verbs can allow secondary imperfectivisation (see Section 2.2.3).

2 Since morphophonological information will be highly relevant, all Serbo-Croatian examples are given in IPA.

3 The theme-vowel classes are based on the allomorphs of the theme vowel in the infinitive (first part) and the present tense forms (second part).

4 An analysis of the vowels preceding inflection as theme vowels is also proposed in Gribanova (2015) for Russian, and Simonović and Mišmaš (2023) for Slovenian.

5 This is a slight simplification of the final version of the account, where the theme vowels are represented as consisting of floating consonants, vocalic features and moras.

6 In some approaches, the inner aspect is distinguished from telicity, the former being computed in a quantity projection immediately above the *v*P and the latter as a property of the *v*P (e.g., Arsenijević, 2006). In addition, some authors differentiate telicity, as a property of the *v*P (telic vs. atelic) from lexical aspect, as a property of the verbs alone, referring to the traditional Vendler (1957)’s classes, i.e., accomplishments, achievements, activities, and states; see Rothstein (2016) for a recent overview.

7 The term grammatical aspect focuses its grammatical function, as opposed to the lexical aspect. The term outer aspect implies its base-generated position ‘out of’ the lexical domain (i.e., above the verb, the *v*P, the first phase, depending on the approach), in opposition to the inner aspect. Finally, the term viewpoint emphasises the perspective (looking at the event from outside vs. inside). Paslawska and von Stechow (2003a, 321-322) use the notion of semantic aspect to capture the containment relations between the event time and reference time, while the term morphological aspect is reserved for aspectual morphemes that do not map 1:1 to semantic aspect. E.g., traditional imperfective morphology in Russian is compatible with both imperfective and perfective aspects. Biskup (2023b,a), on the other hand, uses the term morphological aspect as a synonym for grammatical aspect (see Section 2.2.2 below).

8 As we will see below, even these notions are not equivalent in some approaches.

9 Telicity in this sense does not boil down to the lexical aspect understood as Vendlerian classes. See also Braginsky and Rothstein (2008) for arguments that there are, e.g., both imperfective and perfective (atelic and telic in our terms) accomplishments (based on Russian).

10 On our approach, all traditional perfective verbs are telic, in line with Borer (2005), Łazorczyk (2010), Arsenijević (2023), Milosavljević (2023b). In many formal approaches, Slavic perfectivity and telicity are kept apart, as it is argued that some prefixes, such as the delimitative *po-* or inceptive *za-* induce perfectivity but the verbal predicates are still atelic (Borik, 2006, Gehrke, 2008, Fleischhauer & Gabrovska, 2019, among others; see Milosavljević, 2023b for a detailed overview). The rationale is that perfective verbs with the delimitative *po-* can combine with durative adverbials (which diagnose atelic predicates), whereas perfective verbs with the inceptive *za-* mark a starting point of an event and not telos. However, under the approach to telicity as singular atomicity, these predicates are telic. In the case of the delimitative *po-*, durative adverbials (or the contextually specified quantity) specify the atomic unit and the prefix *po-* restricts the predicate to singularity (Milosavljević, 2022, 2023b). As for the inceptive *za-*, the specification of the initial point is enough for atomicity and singularity. These predicates are also telic under the approach to telicity as non-homogeneity in the sense of Borer (2005) that is compatible with our approach (see Section 2.1 for the definition). Namely, as argued in Borer (2005), verbs with the delimitative *po-* are not cumulative under the standard assumption that *po-* denotes a small quantity (as the sum of two small quantities is not necessarily a small quantity), hence they are non-homogeneous. The similar holds for *za-* prefixed verbs: marking the initial boundary of the event is sufficient for non-homogeneity: such predicates are neither divisive nor cumulative.

11 The perfective version is acceptable with the anterior semantics, which needs to be controlled for while judging.

12 While the aorist and imperfectum are standardly analyzed as tenses (see De Swart, 1998, and Todorović, 2016 for an overview), Arsenijević (2013b) and Todorović (2016) argue that they should be analyzed as aspectual forms at least in Serbo-Croatian, as they specify the relation between the event time and reference time, which is an aspectual distinction (see the overview in Section 2.1). Still, there might be variation in this regard, even within Slavic languages (e.g., Todorović, 2016 argues that these forms are aspects in Serbo-Croatian but tenses in Bulgarian).

13 We do not discuss the issue of linearisation, in particular why the head of QP, which is realised as a prefix, does not head-move with other heads. A possible analysis fully compatible with the present model is offered in Quaglia et al. (2022).

14 As overviewed in Section 2.2.3, there are Slavic languages that allow secondary imperfectivisation of both purely perfectivising prefixes and delimitative *po-*. Addressing the nature of this variation goes beyond the scope of the present paper.

15 An anonymous reviewer raises the question as to whether a similar procedure of categorisation can be found in the nominal domain. We are unaware of any analyses in the literature suggesting this kind of process, but there certainly are empirical phenomena calling for it. For instance, in Serbo-Croatian, there are derived mass nouns in  that arguably incorporate pluralised nouns:  ‘ship’ has the augment  in plural forms (e.g.,  ‘ship.nom.pl’) and this augmented base is used in the mass nominalisation  ‘collection of ships’. We are grateful to the reviewer for bringing up this question.

16 This is the only perfective verb whose aspectual counterpart takes  – judging by the 5.300 most frequent verbs in the database WeSoSlaV (Arsenijević et al., 2024).
